# Including a Technical Factor with Physical and In-Game Movement Factors Improves Model Sensitivity When Evaluating Draft Outcome in Elite-Junior Australian Rules Football

**DOI:** 10.3390/sports11030063

**Published:** 2023-03-09

**Authors:** Jacob Jennings, Daniel Wundersitz, Courtney Sullivan, Stephen Cousins, Michael Kingsley

**Affiliations:** 1 Holsworth Research Initiative, La Trobe Rural Health School, La Trobe University, Bendigo, VIC 3552, Australia; 2 La Trobe University Bendigo Pioneers, Bendigo, Bendigo, VIC 3550, Australia; 3 Department of Exercise Sciences, Faculty of Science, University of Auckland, Auckland 1010, New Zealand

**Keywords:** talent identification, team sports, recruitment, performance, draft

## Abstract

Determining characteristics that define talent is critical for recruitment and player development. When developing predictive models, sensitivity is important, as it describes the ability of models to identify players with draft potential (true positives). In the current literature, modelling is limited to a small number of selected variables, and model sensitivity is often poor or unreported. The aim of this study was to determine how a technical factor combined with physical and in-game movement factors affects position-specific model sensitivity when evaluating draft outcome in an elite-junior National Australia Bank (NAB) League population. Physical, in-game movement, and technical involvement data were collated from draft-eligible (18th year) participants in the under 18 boys NAB League competition (n = 465). Factors identified through parallel analysis were used in binomial regression analyses. Models using factor combinations were developed to predict draft success for all-position, nomadic, fixed-position, and fixed&ruck players. Models that best characterised draft success were all-position (physical and technical: specificity = 97.2%, sensitivity = 36.6%, and accuracy = 86.3%), nomadic (physical and technical: specificity = 95.5%, sensitivity = 40.7%, and accuracy = 85.5%), fixed (physical: specificity = 96.4%, sensitivity = 41.7%, and accuracy = 86.6%), and fixed&ruck (physical and in-game movement: specificity = 96.3%, sensitivity = 41.2%, and accuracy = 86.7%). Including a technical factor improved sensitivity in the all-position and nomadic models. Physical factors and physical and in-game movement yielded the best models for fixed-position and fixed&ruck players, respectively. Models with improved sensitivity should be sought to assist practitioners to more confidently identify the players with draft potential.

## 1. Introduction

Australian Rules football (AF) is one of Australia’s most popular sports, with more than 1.6 million participants nationally [1]. An elite-junior talent pathway operates alongside the elite-senior Australian Football League (AFL), providing talent identification (TID) opportunities for players to enter the competition through an end-of-season draft system. The primary objective of the annual AFL National Draft is to promote equity throughout the league. Teams are allocated a number of selections that occur in succession, with earlier draft selections allocated to the previous season’s less successful teams [2,3]. While any age-eligible (≥18th year) player can nominate for the AFL Draft, the majority of drafted players come from Australia’s elite under 18 (U18) competitions. The NAB League is the Victorian state-based under 18 competition and contributes a large proportion of players (54% in 2021 National Draft) to the AFL each year. 

It is widely understood that a team’s performance is determined by the combination of and interplay between many factors (i.e., collective talent, physical, technical, in-game movement, and tactical), although not all can be quantitatively measured [4]. The ability to identify factors that can categorise players, particularly into an exclusive group (i.e., drafted), could have implications for recruitment and coaching staff along the talent pathway [2,3]. When recruiting from the draft, club staff can identify players that possess desirable qualities that would complement their playing list and areas of deficiency they may need to enhance. If the strengths and weaknesses of players are identified to coaches, training can be optimised for that player and strategies put in place to highlight their talent through specific gameplans or positional decisions. Similarly, if a player is identified to be on the verge of being drafted, effort can be targeted toward improving the qualities required to enhance their draft prospects.

In pursuit of improving understanding and reducing subjectivity in TID and recruitment processes, researchers and practitioners have regularly attempted to identify characteristics associated with key performance outcomes or selection measures in various sports [2,5,6]. Statistical modelling is used extensively in basketball [7] and American football [8] and is becoming more popular in the AF literature, particularly in TID and the draft system [2,5,9]. 

Several studies [2,5,6] demonstrate that physical testing outcomes can predict selection or playing status within elite-junior AF populations. An example that provides good predictive performance, with a model sensitivity of 86% (true positive; correctly classified 86% of those that were elite) and specificity of 74% (true negative; correctly classified 74% of those that were sub-elite), was published in 2015 [2]. A more recent study showed distinctive differences in both physical testing and anthropometric measures that characterise players as talent-identified or non-talent-identified at both a U16 and U18 level [5]. 

When predicting transition from elite-junior to elite-senior competition, however, research is scarce. One study classified draft outcome using a combination of in-game movement and technical involvements, reporting that, in the final reduced model, contested possessions (χ^2^ = 15.2, *p* < 0.001) and inside 50 m kicks (χ^2^ = 11.7, *p* = 0.001) were the two significant variables impacting positive draft outcome. It was concluded that technical involvements are an important factor in draft success; however, model accuracy, specificity, or sensitivity were not reported [10].

Most recently, authors have combined anthropometric, physical testing, and in-game movement data to create a multi-faceted physical profile to investigate factors most associated with draft success [4]. Logistic regression models were generated and model performance was reported. Results indicated that physical characteristics (larger anthropometry and better physical testing outcomes) were better associated with positive draft outcome than in-game movement profile; however, model sensitivity was low (12–38%) and the authors suggested that the inclusion of detailed technical involvement data might enhance the performance of future models. 

When evaluating the literature, several limitations arise that can impact the practical application of findings. Firstly, the breadth of included data has been limited because predictive models have typically only incorporated one or two data sources and few variables even though physical, anthropometric, in-game movement, technical, and psychological variables are often routinely collected in the modern game. Secondly, some conclusions are made without reporting model performance (i.e., sensitivity, specificity, and accuracy). Model sensitivity is particularly important in this context, as it describes a model’s ability to correctly classify true positives (i.e., players with positive draft outcome), which are the players that recruiters are looking to identify. Thirdly, the inclusion of technical involvements in previous modelling has traditionally been limited to a small number of selected variables (e.g., disposals, possessions, and clearances) [3,10], sometimes with no indication of skill outcome (i.e., was the outcome of the involvement positive or negative) [11]. Finally, most of the relevant literature to date uses data obtained prior to 2014. If modelling is to be optimised within the AF literature and application of practical findings improved, the stated limitations need to be considered.

Therefore, using the most current league-wide data collected over three seasons (2017, 2018, and 2019), the aim of this study was to determine how a technical factor combined with physical and in-game movement factors affects position-specific (all-position, nomadic, fixed, and fixed&ruck) model sensitivity when classifying draft outcome in an elite-junior Australian Rules football population.

## 2. Materials and Methods

This study used a retrospective observational cohort design to determine how a player’s physical, in-game movement, and technical involvements contribute to draft outcome. Physical testing data, in-game movement (GPS: Optimeye X4/S5; Catapult Innovations, Melbourne, Australia), and technical involvement data (Champion Data^TM^, Melbourne, Australia) were collated from 12 of the 18 male U18 Australian football teams that competed in the AFL’s NAB League competition during three consecutive seasons (2017, 2018, and 2019). Data were collected as standard procedure during weekly competition and collated and archived at each respective season’s end by the AFL. Data were made available by the AFL for this manuscript and filtered to include only those participants that were in their 18th year and eligible for the AFL National Draft. A player can play in the U18 competition at 16 or 17 years of age but are not eligible for the AFL National Draft until their 18th year. In-game movement and technical involvement data were collected for each player across the season, averaged, and combined with physical testing data in a custom Microsoft Excel spreadsheet (Microsoft Corporation, Washington, DC, USA). The final analyses included 465 participants, of which 90 were drafted and 375 were not. Institutional ethics approval was granted by La Trobe University Human Ethics Committee (ref: HEC20065). 

League-wide physical testing data were collected in March of each year and included the following tests (in order): stature (cm), body mass (kg), standing reach (cm), vertical jump (cm), running vertical jump off each foot (RVJL and RVJR; cm), 20 m sprint with 5 and 10 m splits (s), AFL Agility test (s), and the Yo-Yo Intermittent Recovery Test Level 2 (estimated $\dot{V}O_{2}$ max). Estimated $\dot{V}O_{2}$ max was used due to a different shuttle test being used in 2017, converting distance covered in both tests to a global outcome measure. To ensure a better result was expressed in a greater value, 20 m sprint time was converted to an average speed (m·s^−1^) and AFL Agility time was converted to a positive value using a reverse scoring technique [12]. All testing was conducted indoors on a wooden sprung surface at the conclusion of preseason. In-game movement data were collected using global positioning systems (Optimeye X4/S5; Catapult Innovations, Melbourne, Australia) and included relative distance (m·min^−1^), high-speed running (HSR) efforts, sprint efforts, field time (min), and odometer (m) [4]. High-speed running was classified as 4.00 to 5.99 m·s^−1^, while sprinting was classified as ≥6.00 m·s^−1^. In-game technical involvements were recorded for all players by Champion Data^TM^ to include the technical involvement type and the timestamp at which it occurred, and a single file was provided for the season. Inter-rater reliability of Champion Data^TM^ variables has been externally quantified and deemed acceptable [13]. Technical involvements were grouped to include involvements (n·min^−1^), disposals (n·min^−1^), possessions (n·min^−1^), pressure acts (n·min^−1^), and positive involvements (n·min^−1^). Players were identified as nomadic (midfielders, small and medium defenders, and small and medium forwards), fixed-position (tall forwards and tall defenders), and ruckmen [14]. Given the low number of drafted ruckmen (n = 7), a fixed&ruck (i.e., tall forwards, tall defenders, and ruckmen) group was created for subsequent analyses. 

Data were analysed using the Statistical Package for Social Sciences (version 26, IBM, Armonk, NY, USA). An exploratory factor analysis (principal components analysis with oblique rotation) was conducted prior to binomial logistic regression to identify latent factors and reduce the impact of highly correlated variables. Parallel analysis was used to determine the number of factors that should be extracted from each set (physical, in-game movement, and technical) of data [4]. A suppression threshold of <0.40 was used to identify the variables that were excluded from factors that were used in subsequent analyses [15]. 

Extracted factors and individual variables that did not surpass the minimum loading threshold (positive AFL Agility and estimated $\dot{V}O_{2}$ max) were used in binary logistic regression models to explore relationships with draft status. The Akaike information criterion (AIC) was used as a means for model selection [16]. Analyses were conducted on all positions collectively (all-position) and for each of the three positional groups. Draft outcome was coded as the binomial response variable (1 = drafted, 0 = not drafted), with factors used as the explanatory variables. Best performing models were assessed using model accuracy (correct assessments/all assessments), specificity (true not drafted/all not drafted), and sensitivity (true drafted/all drafted). Odds ratios for coefficients within each best performing model were reported. Statistical significance was set at *p* < 0.05.

## 3. Results

Based on parallel analysis of the combined data set, six factors were extracted (Table 1). Three factors emerged from physical testing variables (speed, anthropometry, and jump), two from in-game movement variables (running effort and contribution) and one from the technical involvement variables (technical). Estimated $\dot{V}O_{2}$ max and AFL Agility loaded on no particular factor and are not presented in Table 1. Table 2 presents regression model accuracy, sensitivity, and specificity for each positional group and the associated factor combinations. 

Unstandardised estimated regression weights and odds ratios for the eight factors are presented in Table 3. For all positions, more technical involvement (OR = 2.52), larger anthropometry (OR = 2.07), the ability to jump higher (OR = 1.73), and a greater estimated $\dot{V}O_{2}$ max (OR = 1.06) significantly increased the odds of being drafted. As a nomadic player, more technical involvement (OR = 3.00), the ability to jump higher (OR = 1.77), and a greater estimated $\dot{V}O_{2}$ max (OR = 1.19) contributed significantly. Larger anthropometry (OR = 8.93) and the ability to jump higher (OR = 4.53) were the significant predictors of being drafted for fixed-position players. Finally, within the fixed&ruck model, AFL Agility (OR = 40.08) contributed greatest, whilst anthropometry (OR = 6.71) and jump ability (OR = 2.83) contributed to a lesser degree.

## 4. Discussion

The aim of this study was to determine how a technical factor combined with physical and in-game movement factors affects position-specific (all-position, nomadic, fixed, fixed&ruck) model sensitivity when classifying draft outcome in an elite-junior Australian Rules football population. Although still only moderately successful, including the technical factor improved the sensitivity of the all-position and nomadic models when compared to just physical and in-game movement models (sensitivity 36.6% and 41.7% vs. 12.7% and 11.1%). The technical factor did not feature in the best performing models for the fixed or fixed&ruck positions. Physical factors were the most prominent, featuring in all best performing models, and were most influential in the fixed and fixed&ruck models, respectively. 

Model sensitivity is an important consideration when trying to identify players with draft potential. High sensitivity reflects a model successfully classifying a high percentage of drafted players. Conversely, high specificity reflects a model’s ability to correctly classify a high percentage of not-drafted players. Model accuracy is the product of correct classifications over all classifications. Proportionately low drafted numbers (NAB League = 15%) mean that, if the accuracy of two models is equal, a model with high sensitivity and specificity has greater application than one with very low sensitivity and very high specificity. Apart from the fixed&ruck group, including the technical factor generated models with greater sensitivity when compared to models that only used combinations of physical and in-game movement factors. More specifically, the addition of the technical factor improved the all-position model sensitivity from 12.7% (physical and in-game movement) to 36.6% (physical and technical). These findings are congruent with previously established associations between the number and type of technical involvements with draft status [10] and draft round order [3]. As elite-junior programs aim to provide a pathway into the elite-senior game, technical development should be an important objective for coaching staff.

Physical factors contributed significantly to all best performing models, where better performance in the variables that combine to form the physical factors significantly increased a player’s odds of being drafted. In the all-position model, greater anthropometry (OR = 2.07), jump performance (OR = 1.73), and estimated $\dot{V}O_{2}$ max (OR = 1.06) were significant contributing factors. The importance of a nomadic player’s jump performance (OR = 1.77) and estimated $\dot{V}O_{2}$ max (OR = 1.19) is congruent with their requirement to cover large distances transitioning between offense and defence, while also contributing to the contest [13]. Nomadic players have been reported to cover 10.5 ± 1 km in an NAB League match and have upwards of 40 technical involvements (many of which will be arial contests), while, in an AFL match, players can cover 12.6 ± 2.2 km with more than 60 technical involvements [17]. Fixed-position players with greater anthropometry (OR = 8.93) and jump performance (OR = 4.53) were more likely to be drafted. Being a bigger (taller and heavier) player with the ability to jump higher than an opposition player allows cleaner marking opportunities for these positions. Both greater anthropometry (OR = 6.71) and jump performance (OR = 2.83) contributed to the fixed&ruck model; however, including ruckmen in the group also resulted in greater agility, increasing the odds of being drafted 40-fold. These results extend previous work that has investigated physical testing outcomes contributing to higher selection status within elite-junior populations [2,3] and more recent work investigating the combination of physical and in-game movement factors associated with draft outcome [5]. Elite-junior programs might consider selecting players that are larger in stature, can jump high, and have good aerobic capacity, while also having access to qualified staff that can continue to develop these characteristics alongside technical skill. 

Although in-game movement factors (running effort and contribution) contributed to the best performing model for fixed&ruck players, neither factor significantly increased the odds of being drafted in that model. This finding is in contrast to one previous study indicating that specific in-game movement variables, such as high-speed distance and relative distance, are associated with the round in which a player is drafted [6], while another showed only trivial to small effects when comparing in-game movement variables between drafted and non-drafted players [8]. These findings could suggest one of two things: firstly, recruiters may not be using the available in-game movement data to its potential and, secondly, given equipment limitations in elite-junior competitions, players being monitored with GPS typically already have recruiter interest, and in-game movement profiles of these players may not differ enough to distinguish between drafted and not-drafted players.

There are several limitations present in this study. Firstly, limitations arise when analysing these retrospective data, including assumptions that GPS setup procedures were the same across teams and reports collated correctly; physical testing data were consistently collected year-on-year; and the position the player was assigned is the position in which they played most of their football during the season. Secondly, the cross-sectional nature of the physical testing data must be considered. It is collected at a single time-point in March each year and then analysed alongside longitudinal in-game movement and technical data. Authors agreed the larger population tested in March compared to those tested just prior to the draft served analysis power favourably. Finally, it is important to consider the large imbalance that existed between drafted and not-drafted players, which might have influenced binomial regression results. Unfortunately, this is a reality of any talent pathway in that participant numbers decrease as players get closer to elite-senior transition.

## 5. Conclusions

This was the first study in AF to combine physical, in-game movement, and technical factors to provide position-specific insight into combinations associated with draft success. When the technical factor was included, more sensitive all-position and nomadic models were generated than when just physical and in-game movement factors were used. Physical factors alone and physical and in-game movement factors provide the best performing models for fixed-position and fixed&ruck players, respectively. Results from this study have application when evaluating a player’s suitability for a particular position, the likelihood that a player will be drafted in that position, and in guiding development of characteristics within elite-junior talent that recruiters deem important. Characteristics of an NAB League player should as closely as possible reflect those of previously drafted players, and elite-junior AF programs should be aiming to develop the physical qualities that underpin running, jumping, and change of direction performance, in addition to position-specific technical ability. Continuing to aim for increased model sensitivity, future research should look to compare outcomes from methods employed in this study and outcomes from more advanced modelling techniques, such as machine learning.

## Figures and Tables

**Table 1:** Factor analysis using physical testing, in-game movement, and technical data.

Variable	Speed	Anthro	Jump	Running Effort	Running Contribution	Technical Involvement
Speed5	0.986					
Speed10	0.984					
Speed20	0.868					
Height		0.963				
Reach		0.923				
Mass		0.877				
VJ			0.890			
RVJL			0.825			
RVJR			0.803			
HSR Efforts				0.925		
m·min^−1^				0.912		
Sprint Efforts				0.653		
Field Time					0.941	
Odometer				0.469	0.824	
Relative Possessions						0.969
Relative Involvements						0.968
Relative Positive						0.965
Relative Disposals						0.953
Relative Pressure Acts						0.536

Note: only factor loadings >0.40 are presented. Positive agility and estimated $\dot{V}O_{2}$ max did not load on any factor, so were treated as additional physical variables. Speed5—5 m speed, Speed10—10 m speed, Speed20—20 m speed, VJ—vertical jump, RVJL—running vertical jump left foot, RVJR—running vertical jump right foot, HSR—high-speed running.

**Table 2:** Binomial logistic regression model performance.

Position	Model	Accuracy (95% CI)	Specificity (95% CI)	Sensitivity (95% CI)	χ^2^ (df)	R	*p*-Value
All-position	Physical	83.5 (79.5–87.0)	98.8 (96.9–99.7)	12.7 (6.0–22.7)	50.15 (5)	0.194	<0.001
	In-game Movt.	80.6 (76.8–84.1)	100.0 (99.0–100.0)	0.0 (0.0–4.0)	13.02 (2)	0.044	0.001
	Tech	80.3 (76.4–83.9)	96.5 (94.0–98.1)	14.4 (7.9–23.4)	57.08 (1)	0.186	<0.001
	Physical + In-game Movt.	82.0 (77.9–85.6)	97.0 (94.5–98.5)	12.7 (6.0–22.7)	56.81 (7)	0.218	<0.001
	**Physical + Tech**	**86.3 (82.5–89.5)**	**97.2 (94.8–98.7)**	**36.6 (25.5–48.9)**	**84.44 (6)**	**0.316**	**<0.001**
	In-game Movt. + Tech	80.8 (76.9–84.3)	96.2 (93.7–97.9)	17.8 (10.5–27.3)	63.60 (3)	0.206	<0.001
	Physical + In-game Movt. + Tech	85.5 (81.6–88.8)	96.3 (93.6–98.1)	36.6 (25.5–48.9)	86.75 (8)	0.324	<0.001
Nomadic	Physical	82.8 (78.0–86.9)	98.0 (95.4–99.3)	13.0 (5.4–24.9)	38.23 (5)	0.195	<0.001
	In-game Movt.	80.3 (75.8–84.3)	100.0 (98.7–100.0)	0.0 (0.0–5.1)	13.63 (2)	0.060	0.001
	Tech	79.9 (75.4–84.0)	95.3 (92.2–97.5)	18.6 (10.3–29.7)	53.42 (1)	0.224	<0.001
	Physical + In-game Movt.	81.8 (77.0–86.0)	97.2 (94.3–98.9)	11.1 (4.2–22.6)	42.11 (7)	0.214	<0.001
	**Physical + Tech**	**85.5 (80.9–89.3)**	**95.5 (92.0–97.7)**	**40.7 (27.6–55.0)**	**74.80 (6)**	**0.364**	**<0.001**
	In-game Movt. + Tech	79.7 (75.1–83.8)	95.0 (91.7–97.2)	18.6 (10.3–29.7)	57.97 (3)	0.242	<0.001
	Physical + In-game Movt. + Tech	85.1 (80.6–89.0)	95.0 (91.5–97.4)	40.7 (27.6–55.0)	74.82 (8)	0.364	<0.001
Fixed	**Physical**	**86.6 (76.0–93.7)**	**96.4 (87.5–99.6)**	**41.7 (15.2–72.3)**	**16.02 (5)**	**0.349**	**0.007**
	In-game Movt.	82.7 (72.2–90.4)	100.00 (94.2–100.0)	0.0 (0.0–24.7)	1.07 (2)	0.024	0.585
	Tech	82.4 (71.8–90.3)	96.7 (88.7–99.6)	15.4 (1.9–45.5)	8.907 (1)	0.187	0.003
	Physical + In-game Movt.	85.1 (74.3–92.6)	96.4 (87.5–99.6)	33.3 (9.9–65.1)	17.09 (7)	0.369	0.017
	Physical + Tech	86.4 (75.7–93.6)	96.3 (87.3–99.6)	41.7 (15.2–72.3)	21.28 (6)	0.450	0.002
	In-game Movt. + Tech	82.4 (71.8–90.3)	96.7 (88.7–99.6)	15.4 (1.9–45.5)	9.21 (3)	0.193	0.027
	Physical + In-game Movt. + Tech	84.8 (73.9–92.5)	96.3 (87.3–99.6)	33.3 (9.9–65.1)	21.79 (8)	0.459	0.005
Fixed&Ruck	Physical	86.7 (78.4–92.7)	98.8 (93.3–100.0)	29.4 (10.3–56.0)	20.43 (5)	0.312	0.001
	In-game Movt.	81.8 (73.3–88.5)	100.0 (96.0–100.0)	0.0 (0.0–16.8)	1.78 (2)	0.026	0.410
	Tech	82.6 (74.1–89.2)	100.0 (95.9–100.0)	5.0 (0.1–24.9)	8.48 (1)	0.122	0.004
	**Physical + In-game Movt.**	**86.7 (78.4–92.7)**	**96.3 (89.6–99.2)**	**41.2 (18.4–67.1)**	**25.12 (7)**	**0.375**	**0.001**
	Physical + Tech	85.6 (77.0–91.9)	97.5 (91.3–99.7)	29.4 (10.3–56.0)	21.24 (6)	0.325	0.002
	In-game Movt. + Tech	79.8 (71.1–86.9)	96.6 (90.5–99.3)	5.0 (0.1–24.9)	11.53 (3)	0.163	0.009
	Physical + In-game Movt. + Tech	85.6 (77.0–91.9)	96.3 (89.4–99.2)	35.3 (14.2–61.7)	26.24 (8)	0.392	0.001

Note: bolded values indicate the best performing model for each positional group.

**Table 3:** Unstandardised estimated regression weights and odds ratios for each position’s best performing model.

Position	Best Performing Model	SE	Lower	OR	Upper
All-position	Physical + Technical				
	Anthro	0.19	1.41	2.07	3.02
	Jump	0.17	1.25	1.73	2.40
	Agility	-	-	-	-
	$\mathrm{Estimated}\;\dot{V}O_{2}$ max	0.05	1.17	1.06	1.29
	Speed	-	-	-	-
	Technical	0.17	1.82	2.52	3.49
Nomadic	Physical + Technical				
	Anthro	-	-	-	-
	Jump	0.20	1.21	1.77	2.60
	Agility	-	-	-	-
	$\mathrm{Estimated}\;\dot{V}O_{2}$ max	0.06	1.06	1.19	1.34
	Speed	-	-	-	-
	Technical	0.20	2.03	3.00	4.45
Fixed	Physical				
	Anthro	0.94	1.43	8.93	55.80
	Jump	0.58	1.47	4.53	13.97
	Agility	-	-	-	-
	$\mathrm{Estimated}\;\dot{V}O_{2}$ max	-	-	-	-
	Speed	-	-	-	-
Fixed&Ruck	Physical + In-game Movt.				
	Anthro	0.75	1.55	6.71	29.06
	Jump	0.40	1.30	2.83	6.16
	Agility	1.26	3.42	40.08	469.73
	$\mathrm{Estimated}\;\dot{V}O_{2}$ max	-	-	-	-
	Speed	-	-	-	-
	Running Effort	-	-	-	-
	Contribution	-	-	-	-

Note: only best performing models and significant (*p* < 0.05) coefficients are presented. Anthro—anthropometry, Estimated $\dot{V}O_{2}$ max —maximal oxygen uptake, Movt.—movement.

## Data Availability

Restrictions apply to the availability of these data as they were obtained from multiple third parties.
